# Enhancement of cauliflower (Brassica oleracea var. botrytis) water stress resistance using paclobutrazol and partial root-zone irrigation

**DOI:** 10.1038/s41598-026-43596-4

**Published:** 2026-05-05

**Authors:** Ahmed F. El-Shafie, Marwa M. Abdelbaset, Ebtessam A. Youssef, Osama M. Dewedar

**Affiliations:** Water Relations and Field Irrigation Department, National Research Centre, Dokki, 12622 Cairo Egypt

**Keywords:** Drought mitigation, Growth regulators, Soil moisture, Irrigation techniques, Irrigation water productivity, Abiotic, Drought

## Abstract

**Water scarcity is one of the major challenges to sustainable development. This challenge will increase as climate change continues, the world’s population grows and the demand for food increases. Therefore, it is necessary to look for agricultural solutions and practices to increase food production while using less water. The study was conducted to investigate the effects of partial root-zone irrigation (50% PRI) and paclobutrazol (growth regulator, PBZ) on the cauliflower crop. This study aims to increase the water stress resistance of the cauliflower crop. Therefore, paclobutrazol was applied 20 and 40 days after planting with different concentrations (0, 25, 50, 75 ppm). In addition, traditional drip irrigation (applying 100% of the irrigation requirement) was used for comparison to 50% PRI under sandy loam soil conditions. The results indicated that the soil moisture content before irrigation increased with increasing paclobutrazol concentration under traditional drip irrigation and the 50% PRI method compared to the control (0 ppm paclobutrazol). Conserving soil water is likely due to the use of paclobutrazol, which may reduce the rate of transpiration from plants. Although some growth characteristics were reduced, plants showed no obvious signs of wilting under 50% PRI with paclobutrazol, as soil moisture content was maintained within available water limits. Moreover, cauliflower yield under 50% PRI with 75 ppm paclobutrazol showed a slight decrease just 1.06% and 1.75% lower than yields achieved under traditional drip irrigation with 0 ppm paclobutrazol during the two growing seasons, respectively. On the other hand, the highest irrigation water productivity was achieved with 50% PRI and 75 ppm paclobutrazol, which showed an increase of approximately 49% compared to traditional drip irrigation without paclobutrazol. These results indicate that combining 50% partial root-zone irrigation (PRI) with 75 ppm paclobutrazol application may achieve comparable yields to traditional drip irrigation (100% water requirement), while reducing irrigation water use by 50%. Finally, as we address future changes in water availability, it is important to develop a long-term water management strategy.**

## Introduction

Climate change has a detrimental influence on the agricultural sector and various aspects of agricultural growth, particularly in developing nations^1,2^. Climate change exacerbates abiotic stressors such as severe drought/waterlogging, temperature extremes, salinity/alkalinity, and abrupt rainfall patterns, all of which have a wide range of effects on plants^3^. Therefore, it is essential to understand the anticipated climate change and its potential effects on agriculture to research future global food and water security and implement all feasible countermeasures^4^. These climate-induced pressures directly threaten global food security. By impairing crop yields and reducing the reliability of harvests, climate change jeopardizes the stable availability of food^5^. This is especially critical for developing nations, where agriculture forms the backbone of both livelihoods and food supply, increasing the risk of malnutrition and hunger in vulnerable populations^6,7^.

Cauliflower (*Brassica oleracea* var. botrytis L.) is a staple vegetable crop worldwide, especially in the Mediterranean region^8,9^. It has medicinal and physiological properties, making it an essential part of the human diet^8,10^. The total area of cauliflower production in Egypt was approximately 4,245 hectares during 2022, with a total production of 114,168 t and an average of 26.89 t ha^− 1^ (FAOSTAT 2022)^11^. Cauliflower is a moderate water consumer compared to other vegetable crops, but its exact water requirements depend on climate, growth stage, and irrigation practices. It requires consistent moisture, especially during head formation^12,13^.

On the other hand, the water crisis is considered one of the greatest problems limiting plant growth and productivity under climate change conditions, especially in arid and semiarid regions^14–17^. As a result, while addressing future changes in water availability, creating a long-term water management strategy is critical. For this reason, it is necessary to choose the optimal and highly efficient irrigation method to save water^18,19^.

Subhan et al.^20^ concluded that the highest irrigation water use efficiency of cauliflower (354.35 kg ha^− 1^ mm^− 1^) was observed under 40% deficit irrigation. Moderate or severe deficit irrigation during juvenility can maintain cauliflower yields at levels similar to full irrigation, improving irrigation water use efficiency (IWUE) without reducing revenue^8^. These strategies are recommended for sustainable water management in cauliflower production. Several deficit irrigation solutions have been developed (e.g., partial root-zone drying (PRD) or, as suggested in the title, partial root-zone irrigation (50% PRI)^21,22^.

The PRD, or PRI 50%, has demonstrated good benefits for both water conservation and water productivity improvement in arid and semiarid locations^23^. The use of partial root-zone drying (PRD) reduced irrigation water usage by approximately 50% in a peach orchard. Although this water-saving strategy (PRD 50%) led to a 20% decrease in yield, the significant reduction in water requirements may render this trade-off acceptable in water-scarce conditions^24^. Drip irrigation enables this by allowing for precise deficit irrigation that minimizes damage from water stress^25^. Therefore, irrigation management involves controlling the amount of time and frequency with which irrigation is applied to maximize its efficiency and minimize plant stress^26,27^. Furthermore, if the intervals between irrigations are regulated automatically, this reduces the waste of water used while providing water to plants easily in real time^28^. In addition, crop water requirements must be met without harming plants^29^.

Water stress, as an abiotic stress, may directly reduce crop growth, possibly through decreased cell elongation, cell turgor, or cell volume, thereby hindering nutrient transfer^30,31^. Paclobutrazol (PBZ), a synthetic plant growth regulator, is a triazole-type inhibitor of gibberellin (GA) biosynthesis that affects plant growth and development. It inhibits the activity of ent-kaurene oxidase, which is an enzyme in the GA biosynthetic pathway that catalyzes the oxidation of ent-kaurene to ent-kaurenoic acid^32^. It acts by inhibiting gibberellin biosynthesis, reducing internodal growth to yield stouter stems, increasing root growth, causing early fruit set, and increasing seed set in plants. When gibberellin production is inhibited, cell division still occurs, but new cells do not elongate, resulting in stouter stems and short internodes with the same number of leaves^33^. The treatment with PBZ brought about significant modifications in cauliflower growth, yielding plants that were more compact, with shorter stems, smaller leaves, a darker green appearance (visually assessed), and earlier flowering^34^. Paclobutrazol acts as a stress protectant by maintaining the relative water content, membrane stability index, photosynthetic activity, and photosynthetic pigments. It also protects the photosynthetic machinery by increasing the level of osmolytes, antioxidant activities, and levels of endogenous hormones, thereby increasing yield^35^.

Therefore, the first objective of this study was to investigate water savings via the partial root-zone irrigation (PRI 50%) method compared to traditional drip irrigation. The second objective was to evaluate the effect of different concentrations of paclobutrazol (0, 25, 50, and 75 ppm) as a stress protectant on the growth and yield of cauliflower under PRI 50% compared to traditional drip irrigation in sandy loam soil.

## Materials and methods

### Experimental site and plant material

Two field trials were carried out in sandy loam soil over the 2022 and 2023 growing seasons. The experimental site is located in Bilbeis city on the southern Nile Delta, Sharqia Governorate, Egypt (latitude 30°22′04.9′′ N, longitude 31°37′38.2′′ E, and mean altitude 21 m above sea level). Some soil physical properties of the experimental site were measured on-site and in the laboratory at the beginning of the experiment **Table S1** (Supplementary File). Soil samples were collected from various sections of the experimental area at depths of 0–30 cm and 30–60 cm. Samples from the same depth were thoroughly mixed to form a composite sample for each layer. Standard methods were used for soil physical characterization. Particle size distribution was determined by the pipette method^36^. Soil moisture retention characteristics, field capacity (FC), and permanent wilting point (PWP) were measured following Gardner^37^. Saturation point (SP) was assessed using the constant head technique^38^.

## Experimental design and layout

**Fig. 1:**
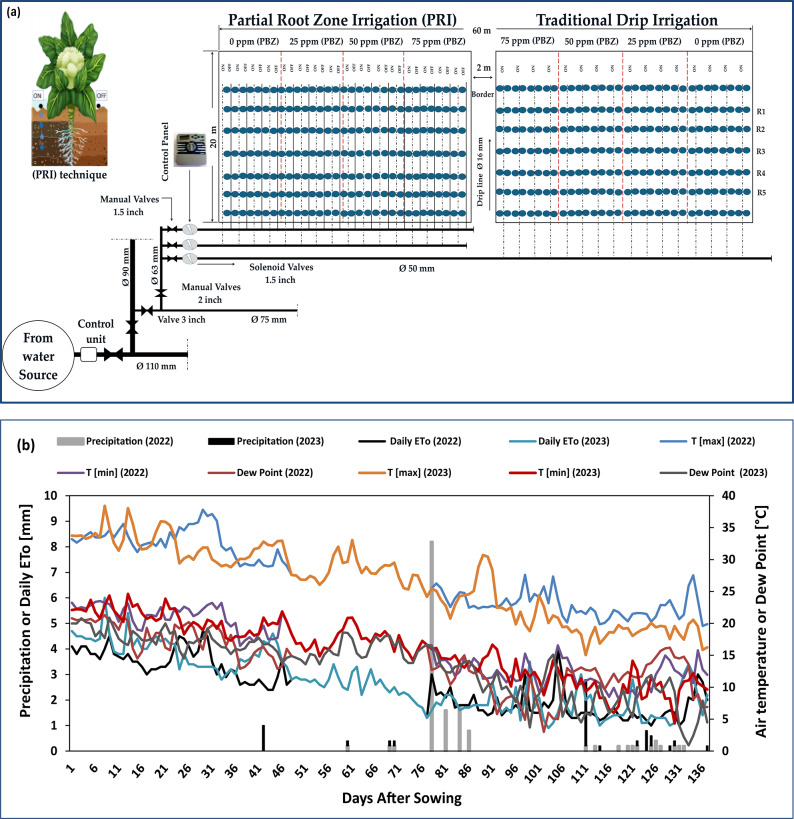
**(a)** Layout of the field experiment, automatic control method for traditional drip irrigation (DI) and partial root-zone irrigation (PRI), distribution of paclobutrazol (PBZ) treatments with concentrations (0, 25, 50, 75 ppm), and number and distribution of replicates (**b**) The daily of meteorological data, daily air temperature (T min and max), dew Point, precipitation and reference evapotranspiration (ETo) for the experimental site of Belbeis region during the two growing seasons (2022 and 2023).

The experimental layout was a split-plot design with five replicates. Replicates were randomly distributed along the drip irrigation lines, with borders between treatments. The main plot (first factor) comprised two irrigation methods (traditional drip irrigation and PRI 50%), and the subplot (second factor) included paclobutrazol concentrations (PBZ) at 25, 50, and 75 ppm, as well as the control (0 ppm). Paclobutrazol concentrations were applied 20 and 40 days after planting. The experiment included 8 treatments distributed in 8 experimental units; the unit area was 145 m^2^. For traditional drip irrigation, each unit has 12 lateral lines, each 20 m in length, with a distance of 0.6 m between lines. Otherwise, for the PRI, each unit contains 24 laterals, each 20 m in length, with a distance of 0.6 m between lines. Figure 1a represents only a section of the layout.

Healthy, uniform-sized cauliflower (*Brassica oleracea* var. botrytis) seedlings were selected from a nursery and transplanted on the first of September (35 days after sowing) in both seasons. The seedlings were transplanted on both sides of the driplines, with a spacing of 0.5 m between plants within the row. To ensure seedling vitality and avoid water stress after transplanting, all plants, regardless of treatment, were irrigated every 2 days. Irrigation treatments began 10 days after planting. Irrigation treatments consisted of two levels: 50% of the crop water requirement applied through Partial Root- zone Irrigation (PRI) and 100% of the water requirement applied using traditional drip irrigation.

## Irrigation systems and scheduling

An automatic irrigation system was installed under all the experimental treatments. The irrigation system included a pump, pressure gauges, a filter, a flow meter, a fertilizer injection unit, a control panel, and solenoid valves. The drip irrigation method was selected, using built-in emitters with an average flow rate of 4.0 L per hour (L h^− 1^) at an operating pressure of 0.1 MPa and a spacing of 0.3 m between emitters. Two techniques were selected via an automated drip irrigation. The first was traditional drip irrigation (full irrigation requirement), with a distance of 0.6 m lateral spacing. The second was partial root-zone irrigation (50% PRI), a type of deficit irrigation (50% of the irrigation requirement). Partial root-zone irrigation is a method of managing moisture and drought at the same time within the root zone. It also specifies that approximately half of the crop’s root zone is irrigated (50% of full irrigation), whereas the other half remains unirrigated. The irrigation lines are switched on/off between alternating sides of the root zone^39,40^. Moreover, there was a difference in the design of the PRI 50% network, where two submain lines were installed, each with a separate valve. The first line was designed with a distance of 0.6 m between the lateral lines. The second submain line was then designed so that the lateral line was installed at the middle distance between every two laterals on the first submain line (Fig. 1a).

In addition, the CROPWAT 2012 program (version 8.0.1.1) was used to calculate the crop water requirement (mm) and schedule irrigation (mm/interval) for the cauliflower crop, based on the study’s soil type and regional climate data. The model implemented the FAO-Penman-Monteith equation of Allen et al.^12^ using meteorological data from 2022 to 2023 (latitude 30°23’55.4"N, longitude 31°34’39.1"E, and mean altitude 21 m above sea level). The daily meteorological data and reference evapotranspiration of the two seasons for the experimental site of the Belbeis region are shown in Fig. 1b. A crop coefficient (Kc) from Allen et al.^41^ was applied to determine the actual evapotranspiration. The gross irrigation requirements were converted from mm/ha/day to m^3^ ha^[-1^ day^[-1^.

The crop water requirements for both irrigation methods were met after cultivation. The water requirements for traditional drip irrigation and PRI 50% were 2200 and 1100 m^3^ ha^− 1^, respectively, and 2420 and 1210 m^3^ ha^− 1^, respectively, for the two growing seasons. The irrigation rate (m^3^ day^− 1^) was converted into time. Accordingly, the actual irrigation time was preset for each irrigation method. In general, the irrigation time under partial root-zone irrigation was approximately half that under traditional drip irrigation. Finally, all the cauliflower plants were subjected to the same horticultural practices except for the experimental treatments.

### Soil moisture content

Soil moisture was measured per treatment in all five replicate blocks using a PR2 probe (Delta-T Devices Co., Cambridge, UK) equipped with four sensor rings. Measurements were taken at key positions relative to the plant root zone: at varying distances from the emitter (0, 5, 10, and 15 cm along the x-axis) to assess moisture distribution patterns under drip irrigation. Additionally, measurements were recorded at different soil depths (10, 20, 30, and 40 cm along the y-axis) to evaluate vertical water movement and root uptake efficiency. The data were analyzed using SURFER software for contour mapping and spatial modeling of soil moisture dynamics in the active root zone.

### Crop growth quality characteristics

Cauliflower plants were harvested at horticultural maturity in both seasons, in mid-January (~137 days after transplanting). To ensure representative sampling, 5 plants per treatment replicate were randomly selected along the drip irrigation line, excluding guard rows (border plants) to minimize edge effects. At harvest, samples were collected to record vegetative growth quality characteristics (plant fresh weight (g), root fresh weight (g), leaf fresh weight (g), and number of leaves per plant), as well as yield parameters (head diameter, head weight, and head-to-plant percentage).

### Leaf total chlorophyll content, total soluble solids percentage and cell sap osmotic pressure

Leaf chlorophyll content was estimated using a portable SPAD-502 chlorophyll meter (Minolta Camera Co., Osaka, Japan). For each plant and treatment, measurements were taken on five leaves, with five replicate readings per leaf^42^.

The total soluble solids percentage (TSS) was determined from leaf cell sap. Sap was extracted from fresh leaf discs using a manual press. The extracted sap was then immediately analyzed using a hand refractometer (Digital Refractometer 0–53% Brix, Model: PAL-53, ATAGO Co., Ltd., Tokyo, Japan). Five replicate leaf samples per treatment were measured.

The leaf cell sap concentration and osmotic pressure (MPa) were determined according to Gusev^43^. Gusev’s Method determines leaf cell sap concentration by extracting sap from frozen-thawed leaf tissue. Osmotic potential was measured using a vapor pressure osmometer (Model VAPRO 5520, Wescor, Inc., Logan, UT, USA).

### Total yield and irrigation water productivity

At harvest, the total weight of the plants in each treatment was recorded as kg per 1 m^2^, after which the total yield was calculated as t ha^− 1^.

The marketable yield divided by applied irrigation is defined as irrigation water productivity “IWP” (kg m^− 3^), Eq. (2) was used as stated by Fernández et al.^44^.

1 $$IWP=\frac{MarketableYield\left(kg{ha}^{-1}\right)}{AppliedIrrigation\left({m}^{3}{ha}^{-1}\right)}$$

### Statistical analysis

The experimental design was a split plot in a completely randomized block design with five replicates. The obtained data were statistically analyzed via the SPSS statistical software program (version 25) and the analysis of variance method as reported previously by Snedecor and Cochran^45^. The differences between means were compared using Duncan’s multiple range test^46^.

## Results

### PBZ-associated soil moisture conservation under PRI

**Fig. 2:**
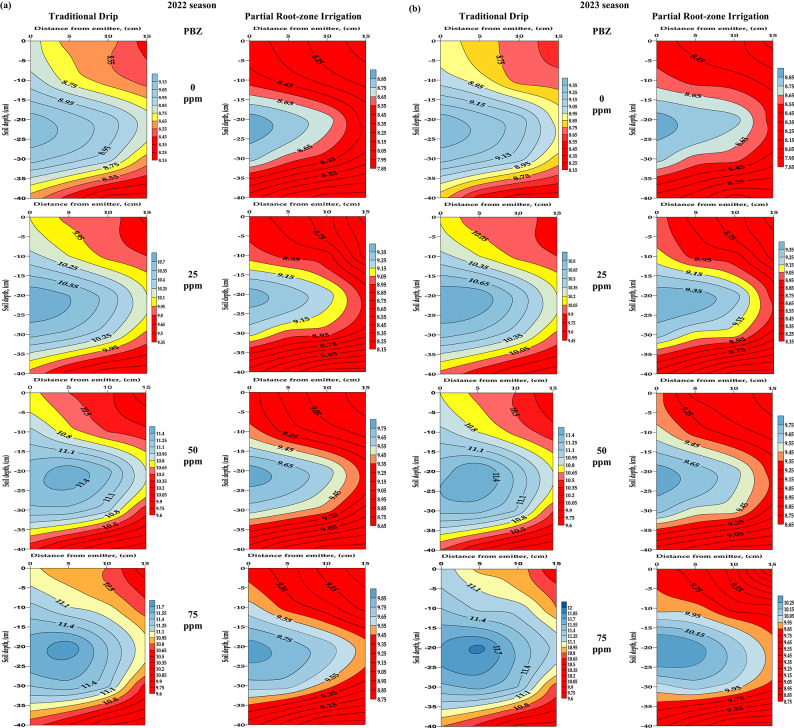
Soil moisture (%) distribution values before irrigation under traditional drip and partial root-zone irrigation (PRI) methods at 55 days after planting after sprayed with different concentrations of paclobutrazol (PBZ) (0, 25, 50, 75 ppm), for (**a**) 2022 and (**b**) 2023 seasons. Measurements were taken at distances from the emitter (0, 5, 10, and 15 cm along the x-axis). Additionally, measurements were recorded at different soil depths (10, 20, 30, and 40 cm along the y-axis). The data were analyzed using SURFER software for contour mapping and spatial modeling. Values are means of 5 replicates ± SE; by Duncan’s new multiple range test at *p* < 0.05.

The spatial distribution of soil moisture under different irrigation systems and paclobutrazol (PBZ) concentrations is presented in Fig. 2a (2022 season) and Fig. 2b (2023 season). Statistical analysis revealed a significant interaction between irrigation method and PBZ concentration (*p* < 0.05), indicating that the effect of PBZ on soil moisture was irrigation-dependent. In both seasons, PBZ application significantly improved soil moisture retention, with the most pronounced effect observed under the water-limited 50% partial root-zone irrigation (PRI) system. Under 50% PRI without PBZ, the lowest average soil moisture values were recorded (8.37% in 2022 and 8.47% in 2023). Conversely, PBZ application mitigated this deficit, with moisture levels increasing progressively with PBZ concentration, reaching 10.02% and 10.12% under 50% PRI with 75 ppm PBZ in 2022 and 2023, respectively. Under traditional drip irrigation (full irrigation), soil moisture was naturally higher across all treatments. The highest average moisture values were achieved with 75 ppm PBZ, reaching 11.08% in 2022 and 11.21% in 2023. The contour maps (Fig. 2a, b) visually confirm these trends, showing broader and more uniform moisture distribution under traditional drip irrigation and a clear mitigation of dry zones under 50% PRI when PBZ was applied. These results indicatethat paclobutrazol potentiallyconserves soil moisture under deficit irrigation, significantly reducing water stress in cauliflower during critical growth stages.

### Growth–quality trade-offs and curd partitioning under deficit irrigation

**Fig. 3:**
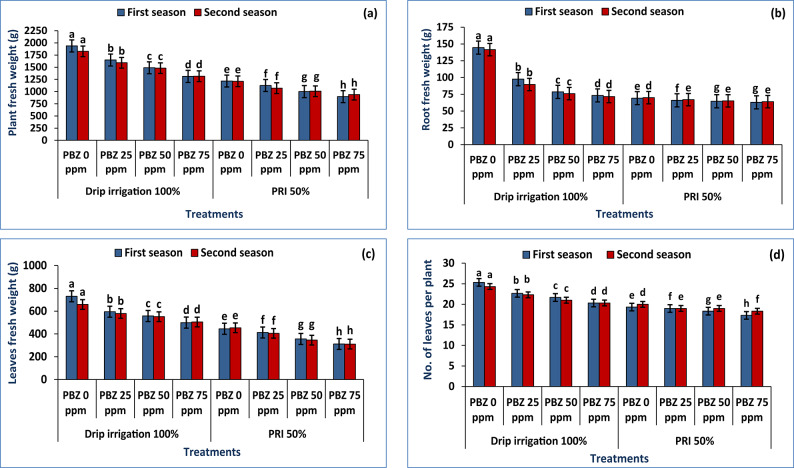
Effects of interaction between irrigation strategy drip irrigation 100% of irrigation requirement (DI), and partial root-zone irrigation (PRI) 50% of irrigation requirement and spring with different concentrations of paclobutrazol (PBZ) 0, 25, 50 and 75 ppm, respectively on some growth characteristics of cauliflower plants (2022 and 2023 seasons). (**a)** Plant fresh weight. (**b)** Root fresh weight. (**c)** Leaf fresh weight. (**d)** Number of leaves per plant. Values are means of 5 replicates ± SE; the same alphabets means that statistically non-significant by Duncan’s new multiple range test at *p* < 0.05.

The results indicated that the paclobutrazol concentration had a negative effect on some growth parameters of the cauliflower crop in both seasons (Table 1**and** Fig. 3). For the irrigation factor, the higher growth parameters (fresh plant weight, fresh root weight, fresh leaf weight, and number of leaves per plant) were recorded under traditional drip irrigation than under 50% PRI for both seasons. For the paclobutrazol factor, the highest value of plant fresh weight was obtained with 0 ppm paclobutrazol, whereas the lowest value was obtained with 75 ppm paclobutrazol for the two seasons (Table 1). The inhibitory effect of the paclobutrazol concentration on cauliflower growth characteristics was more evident with the interaction between the irrigation method and the paclobutrazol concentration (Fig. 3a, b, c, **and d**). For plant fresh weight and leaf fresh weight, a significant interaction between irrigation and PBZ was observed in both seasons. This indicates that the effect of PBZ on reducing biomass was dependent on the irrigation level, with the most substantial growth reduction occurring under the combined treatment of 50% PRI and 75 ppm PBZ (Fig. 3a, b, c, **and d**). In contrast, the interaction for root fresh weight and number of leaves per plant was significant only in the 2022 season. During the 2023 season, the effects of irrigation and PBZ on these two parameters were independent, with no significant interaction term. Despite this seasonal variation, the main effects were clear: 50% PRI consistently reduced values compared to full irrigation, and higher PBZ concentrations (50 and 75 ppm) consistently suppressed growth compared to the 0 ppm control.

**Table 1:** Effects of irrigation strategy drip irrigation 100% of irrigation requirement (DI), and partial root-zone irrigation (PRI) 50% of irrigation requirement and spring with different concentrations of paclobutrazol (PBZ) 0, 25, 50 and 75 ppm, respectively on plant fresh weight (g), root fresh weight (g), leaf fresh weight (g) and number of leaves per plant of cauliflower plants (2022 and 2023 seasons). Values are means of five replicates ± SE. Means within a column followed by the same superscript letter are not significantly different according to Duncan’s new multiple range test at *p* < 0.05. Uppercase letters (A–D) indicate comparisons among main effects.

Treatments	Plant fresh weight (g)		Root fresh weight (g)		Leaves fresh weight (g)		No. of leaves per plant	
	First season	Second season	First Season	Second season	First Season	Second season	First season	Second season
**DI 100%**	1597.30 ± 38.2^A^	1553.90 ± 36.1^A^	98.58 ± 2.5^A^	94.75 ± 2.4^A^	595.58 ± 14.8^A^	573.58 ± 14.2^A^	22.50 ± 0.5^A^	22.00 ± 0.5^A^
**PRI 50%**	1060.00 ± 25.8^B^	1057.10 ± 25.6^B^	65.75 ± 1.7^B^	66.58 ± 1.8^B^	381.42 ± 9.8^B^	379.00 ± 9.7^B^	18.50 ± 0.4^B^	19.08 ± 0.4^B^
**PBZ 0 ppm**	1333.73 ± 32.5^A^	1307.20 ± 31.8^A^	91.67 ± 2.3^A^	90.44 ± 2.3^A^	476.78 ± 12.1^A^	467.11 ± 11.9^A^	20.56 ± 0.5^A^	20.78 ± 0.5^A^
**PBZ 25 ppm**	1190.40 ± 29.2^B^	1160.93 ± 28.5^B^	73.67 ± 1.9^B^	71.44 ± 1.8^B^	417.56 ± 10.7^B^	416.78 ± 10.6^B^	19.44 ± 0.4^B^	19.44 ± 0.4^B^
**PBZ 50 ppm**	1063.07 ± 26.3^C^	1054.67 ± 26.1^C^	65.67 ± 1.7^C^	65.67 ± 1.7^C^	380.78 ± 9.8^C^	372.89 ± 9.6^C^	18.67 ± 0.4^C^	18.89 ± 0.4^C^
**PBZ 75 ppm**	930.40 ± 23.2^D^	960.00 ± 24.0^D^	61.22 ± 1.6^D^	61.78 ± 1.6^D^	330.11 ± 8.6^D^	338.33 ± 8.8^D^	17.56 ± 0.4^D^	18.22 ± 0.4^D^

**Fig. 4:**
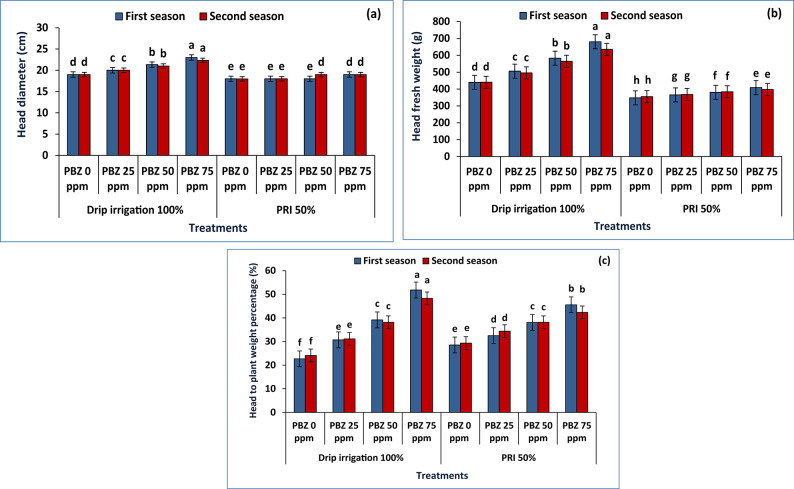
Effects of interaction between irrigation strategy drip irrigation 100% of irrigation requirement (DI), and partial root-zone irrigation (PRI) 50% of irrigation requirement and spring with different concentrations of paclobutrazol (PBZ) 0, 25, 50 and 75 ppm, respectively on some growth characteristics of cauliflower plants (2022 and 2023 seasons). **(a)** The head diameter. **(b)** Head fresh weight. **(c)** Head to plant fresh weight%. Values are means of 5 replicates ± SE; the same alphabets means that statistically non-significant by Duncan’s new multiple range test at *p* < 0.05.

Table 2 shows the effect of irrigation strategy and different concentrations of paclobutrazol. Regarding the irrigation factor, it was noted that the highest head diameter and head weight were obtained under traditional irrigation. While the highest head-to-plant percentage was achieved under partial root-zone irrigation. On the other hand, for the paclobutrazol concentration factor, it was observed that the highest head diameter, head weight, and head-to-plant percentage were obtained under the highest concentration of paclobutrazol (75 ppm). The results revealed that irrigation strategy and paclobutrazol (PBZ) concentration significantly influenced cauliflower yield characteristics, though the significance of their interaction was trait-dependent (Fig. 4a, b, **and c**). A significant interaction between irrigation and PBZ was found specifically for the head-to-plant percentage (Fig. 4c), indicating that PBZ’s effect on biomass partitioning to the marketable curd was dependent on the water regime. For head diameter and head weight, where the interaction was not significant, the main effects were clear. The widest head diameter and heaviest heads were consistently recorded under traditional drip irrigation, while the smallest values were found under 50% PRI. A key finding, however, was that head diameter under traditional drip irrigation (0 ppm PBZ) was statistically equivalent to that under 50% PRI with 75 ppm PBZ (Fig. 4a). This suggests that a high PBZ concentration can partially mitigate the negative impact of water stress on this specific yield component. The data for head weight and biomass partitioning reveals a critical trade-off. As shown in Fig. 4b, head fresh weight under 50% PRI with 75 ppm PBZ was lower than under traditional irrigation with 0 ppm PBZ. Conversely, Fig. 4c demonstrates an inverse pattern for efficiency, where the head-to-plant percentage was significantly higher under PRI with 75 ppm PBZ than under traditional irrigation with 0 ppm PBZ. This points to a strategic balance between water use and yield efficiency. For instance, while traditional irrigation with 75 ppm PBZ yielded the highest absolute head-to-plant percentage, the 50% PRI treatment with 75 ppm PBZ achieved the second-highest value (Fig. 4c) but used only half the amount of water. This combination of deficit irrigation and PBZ presents a viable strategy for optimizing water use efficiency while maintaining a highly favorable proportion of marketable yield.

**Table 2:** Effects of irrigation strategy drip irrigation 100% of irrigation requirement (DI), and partial root-zone irrigation (PRI) 50% of irrigation requirement and spring with different concentrations of paclobutrazol (PBZ) 0, 25, 50 and 75 ppm, respectively on the head diameter (cm), head fresh weight and head to plant fresh weight% of cauliflower plants (2022 and 2023 seasons). Values are means of 5 replicates ± SE; the same alphabets means that statistically non-significant by Duncan’s new multiple range test at *p* < 0.05.

Treatments	Head diameter (cm)		Head fresh weight (g)		Head to plant fresh weight% (%)	
	First Season	Second season	First Season	Second Season	First Season	Second season
**DI 100%**	20.83 ± 0.42^A^	20.58 ± 0.41^A^	552.50 ± 13.8^A^	534.00 ± 13.4^A^	34.59 ± 0.86^B^	34.37 ± 0.85^B^
**PRI 50%**	18.25 ± 0.37^B^	18.50 ± 0.38^B^	375.83 ± 9.4^B^	376.33 ± 9.4^B^	36.38 ± 0.91^A^	36.57 ± 0.91^A^
**PBZ 0 ppm**	16.89 ± 0.34^D^	17.00 ± 0.34^D^	331.44 ± 8.3^D^	336.33 ± 8.4^D^	24.85 ± 0.62^D^	25.73 ± 0.64^D^
**PBZ 25 ppm**	17.44 ± 0.35^C^	17.67 ± 0.35^C^	368.00 ± 9.2^C^	368.33 ± 9.2^C^	30.91 ± 0.77^C^	31.73 ± 0.79^C^
**PBZ 50 ppm**	18.44 ± 0.37^B^	18.89 ± 0.38^B^	420.44 ± 10.5^B^	419.11 ± 10.5^B^	39.55 ± 0.99^B^	39.74 ± 0.99^B^
**PBZ 75 ppm**	19.56 ± 0.39^A^	19.44 ± 0.39^A^	472.44 ± 11.8^A^	456.22 ± 11.4^A^	50.78 ± 1.27^A^	47.52 ± 1.19^A^

### Physiological adjustment to water deficit: chlorophyll maintenance and osmotic regulation with PBZ and PRI

**Fig. 5:**
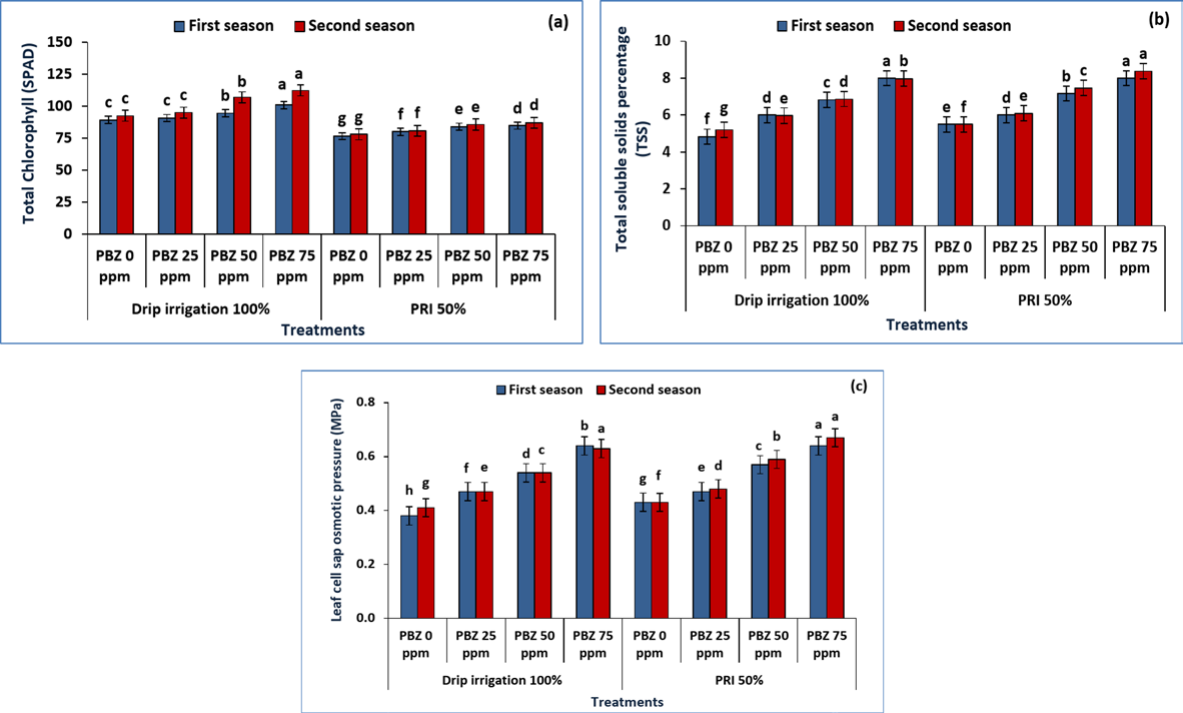
Effects of interaction between irrigation strategy drip irrigation 100% of irrigation requirement (DI), and partial root-zone irrigation (PRI) 50% of irrigation requirement and spring with different concentrations of paclobutrazol (PBZ) 0, 25, 50 and 75 ppm, respectively on some chemical characteristics of cauliflower plants (2022 and 2023 seasons). **(a)** Total chlorophyll. **(b)** Total soluble solids percentage and **(c)** Leaf cell sap osmotic pressure. Values are means of 5 replicates ± SE; the same alphabets means that statistically non-significant by Duncan’s new multiple range test at *p* < 0.05.

The physiological response of cauliflower plants to the interaction between irrigation method and paclobutrazol (PBZ) concentration was significant for total chlorophyll content, total soluble solids (TSS), and leaf cell sap osmotic pressure in both growing seasons (Table 3). The results showed that the highest total chlorophyll content was obtained under traditional drip irrigation for the two seasons, while partial root-zone irrigation (PRI 50%) yielded significantly lower values for the two seasons. Conversely, the 50% PRI strategy induced a stronger osmotic adjustment response, resulting in slightly higher leaf TSS and osmotic pressure compared to full irrigation. Paclobutrazol application consistently enhanced all measured physiological parameters. The 75 ppm concentration produced the highest values for chlorophyll content, as well as for TSS and osmotic pressure. The interaction between these factors was particularly revealing (Fig. 5). The most favorable conditions for chlorophyll accumulation were the combination of traditional drip irrigation with 75 ppm PBZ. In contrast, the most intense osmotic stress response, indicated by the highest TSS and osmotic pressure, was triggered by the combined treatment of 50% PRI and 75 ppm PBZ.

**Table 3:** Effects of irrigation strategy drip irrigation 100% of irrigation requirement (DI), and partial root-zone irrigation (PRI) 50% of irrigation requirement and spring with different concentrations of paclobutrazol (PBZ) 0, 25, 50 and 75 ppm, respectively on the total chlorophyll content, total soluble solids percentage and leaf cell sap osmotic pressure of cauliflower plants (2022 and 2023 seasons). Values are means of 5 replicates ± SE; the same alphabets means that statistically non-significant by Duncan’s new multiple range test at *p* < 0.05.

Treatments	Total Chlorophyll SPAD		Total soluble solids percentage TSS		Leaf cell sap osmotic pressure (MPa)	
	First season	Second Season	First season	Second season	First Season	Second season
**DI 100%**	89.19 ± 2.23^A^	93.84 ± 2.35^A^	6.42 ± 0.16^B^	6.50 ± 0.16^B^	0.50 ± 0.02^B^	0.51 ± 0.02^B^
**PRI 50%**	86.15 ± 2.15^B^	90.84 ± 2.27^B^	6.67 ± 0.17^A^	6.86 ± 0.17^A^	0.52 ± 0.02^A^	0.54 ± 0.02^A^
**PBZ 0 ppm**	76.38 ± 1.91^D^	78.17 ± 1.95^D^	5.44 ± 0.14^D^	5.47 ± 0.14^D^	0.43 ± 0.02^D^	0.43 ± 0.02^D^
**PBZ 25 ppm**	83.32 ± 2.08^C^	85.69 ± 2.14^C^	6.06 ± 0.15^C^	6.18 ± 0.15^C^	0.47 ± 0.02^C^	0.48 ± 0.02^C^
**PBZ 50 ppm**	88.89 ± 2.22^B^	92.97 ± 2.32^B^	7.28 ± 0.18^B^	7.33 ± 0.18^B^	0.58 ± 0.02^B^	0.58 ± 0.02^B^
**PBZ 75 ppm**	95.64 ± 2.39^A^	105.68 ± 2.64^A^	8.39 ± 0.21^A^	8.38 ± 0.21^A^	0.67 ± 0.03^A^	0.67 ± 0.03^A^

### Yield stability and irrigation water productivity under combined PBZ and PRI management

**Fig. 6:**
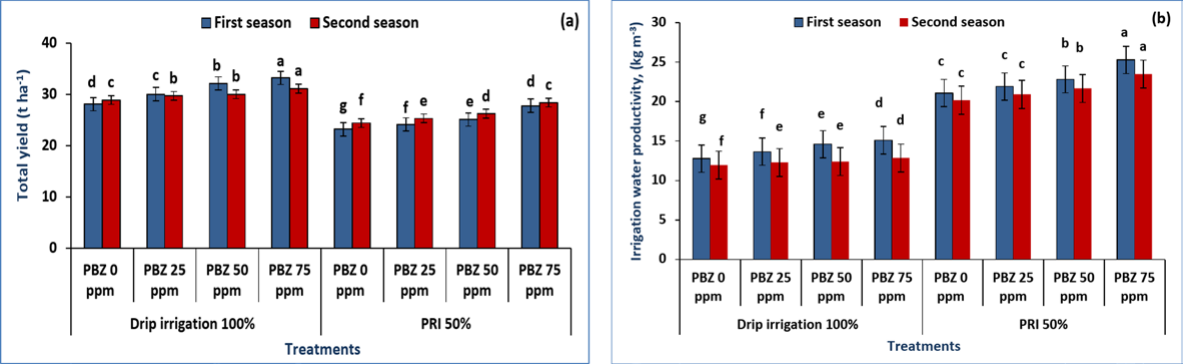
Irrigation strategy drip irrigation 100% of irrigation requirement (DI), and partial root-zone irrigation (PRI) 50% of irrigation requirement and spring with different concentrations of paclobutrazol (PBZ) 0, 25, 50 and 75 ppm, respectively on **(a)** the yield and **(b)** irrigation water productivity of cauliflower crops in the 2022 and 2023 seasons. Values are means of 5 replicates ± SE; the same alphabets means that statistically non-significant by Duncan’s new multiple range test at *p* < 0.05.

The final yield (weight vs. area) of any crop is the most important factor for farmers. The yield results revealed the higher performance of the traditional drip irrigation method with increasing concentrations of paclobutrazol. The highest commercial yields were 33.2 and 31.1 t ha^− 1^ with traditional drip irrigation with 75 ppm paclobutrazol for the two growing seasons, respectively. The lowest yields were obtained under PRI 50% and 0 ppm paclobutrazol (23.2 and 24.4 t ha^− 1^) respectively, for the two growing seasons (Fig. 6a). On the other hand, the yield increased by 21.1% and 18.4% under the traditional drip irrigation method compared with that under the partial root-zone irrigation method for the control treatment during the two growing seasons. Moreover, the results revealed an increase in cauliflower production with increasing paclobutrazol concentration. For the first growing season, the control group (traditional drip irrigation, 0 ppm PBZ) yielded 28.1 t ha⁻¹. The 50% PRI strategy reduced yield to 23.2 t ha⁻¹; however, applying paclobutrazol (PBZ) significantly offset this loss in a dose-dependent manner. Under PRI, yield increased with PBZ concentration, rising from 23.2 t ha⁻¹ (0 ppm) to 27.8 t ha⁻¹ (75 ppm), with the highest PBZ treatment nearly matching the yield of the unstressed control. During the second season, the total yield under 50% PRI was 24.4 t ha⁻¹ without paclobutrazol (0 ppm). Yields increased progressively with higher PBZ concentrations, reaching 25.3, 26.2, and 28.4 t ha⁻¹ with 25, 50, and 75 ppm PBZ, respectively. The yield achieved with 75 ppm PBZ under 50% PRI (28.4 t ha⁻¹) was nearly equivalent to the control yield of 28.9 t ha⁻¹ under traditional drip irrigation with 0 ppm PBZ (Fig. 6a**).** The yields under 50% PRI with 75 ppm paclobutrazol were 27.8 and 28.4 t ha⁻¹ for the two respective seasons. The yield reduction under 50% PRI with 75 ppm paclobutrazol was 1.06% and 1.75% compared to that under traditional drip irrigation with 0 ppm paclobutrazol for the two respective seasons. Thus, the yield achieved under 50% PRI with a high PBZ concentration was comparable to that of the unstressed control using traditional drip irrigation (Fig. 6a).

Irrigation water productivity is an indicator of the relationship between marketable crop productivity in kg ha^− 1^ and the total amount of irrigation applied (m^3^ ha^− 1^) during the crop growing season^44^. Compared with traditional drip irrigation, there was an increase in irrigation water productivity under 50% PRI. The highest values ​​of irrigation water productivity were obtained with 50% PRI and 75 ppm paclobutrazol (25.3 and 23.5 kg m^− 3^), respectively. The lowest values were obtained under 0 ppm paclobutrazol and traditional drip irrigation (12.8 and 11.9 kg m^− 3^, respectively) for the two growing seasons. An increase in irrigation water productivity was achieved by applying 50% PRI with 75 ppm paclobutrazol (49.4 and 49.3%) compared with traditional drip irrigation with 0 ppm paclobutrazol for both seasons (Fig. 6b).

## Discussion

### Mechanisms of PBZ-mediated soil moisture conservation under PRI

The results in Fig. 2 demonstrate that paclobutrazol application increased soil moisture content under both irrigation systems across both growing seasons. In both irrigation treatments, with increasing paclobutrazol concentration, the soil moisture content increased; thus, the plants were not exposed to more water stress compared with those in the control. The increase in the soil moisture values of the paclobutrazol-treated plants compared with those of the control plants was due to several factors. Paclobutrazol is a growth regulator that modulates hormonal balance and growth, which leads to increased tolerance to abiotic stress and improved physiological characteristics of crops^32,47^. It acts by inhibiting gibberellin biosynthesis, reducing internodal growth to yield stouter stems, increasing root diameter, reducing length, and reducing lateral root growth. This causes early fruit set and increases plant seed formation. Additionally, when gibberellin production is inhibited, cell division still occurs, but new cells do not elongate.

The increased soil moisture under higher paclobutrazol (PBZ) concentrations in both irrigation treatments (Fig. 2) can be primarily explained by a reduction in plant water use. Our data show that PBZ application significantly reduced vegetative growth, including plant fresh weight and leaf number (Table 1; Fig. 3). This reduction in canopy size directly decreases the plant’s transpiration surface area. This morphological effect is consistent with PBZ’s known biochemical action as a gibberellin biosynthesis inhibitor, which suppresses stem and leaf expansion, resulting in a compact plant architecture^32,47,48^. This physiological adaptation was complemented by the 50% partial root-zone irrigation (PRI) strategy, which itself inhibited growth compared to traditional drip irrigation (Table 1; Fig. 3). While our study did not measure specific chemical signals, the well-documented PRI response involves root-generated signals that promote conservative water use^49,50^. Consequently, despite reduced growth, plants under the combined treatment showed no wilting, as soil moisture was maintained within adequate limits. Therefore, the combined effect of PBZ (reducing canopy size) and PRI (inducing a conservative growth and water use pattern) synergistically lowered overall plant water demand. This synergy is directly reflected in the conserved soil moisture levels observed under the combined 50% PRI and PBZ treatment (Fig. 2).

### Interpreting growth suppression versus enhanced economic yield partitioning

The results showed that paclobutrazol (PBZ) application consistently reduced vegetative growth parameters, including plant fresh weight, root fresh weight, and leaf number, as detailed in Table 1. This aligns with the known function of PBZ as a growth retardant that inhibits gibberellin biosynthesis, leading to more compact plants with darker green leaves^47,48^. The reduction in leaf area is a key adaptive trait, as it likelyhelps reduce transpiration water loss and improves water use efficiency^32,33^. The combination of PBZ and the 50% partial root-zone irrigation (PRI) strategy induced a synergistic water-saving effect. This enhanced resilience was clearly reflected in the yield characteristics. The results revealed a significant influence of irrigation, PBZ, and their interaction on yield components such as head diameter and head-to-plant percentage, as shown in Fig. 4; Table 2. A key finding was that it was possible to achieve a head diameter statistically equivalent to the well-watered control (Fig. 5a) while saving 50% of the irrigation water through the PRI strategy combined with the application of 75 ppm paclobutrazol at 20 and 40 days after planting. This demonstrates a successful mitigation of water stress on a critical yield component. Furthermore, the data reveals a strategic trade-off: while absolute head weight was lower under water stress (Fig. 4b), the head-to-plant percentage, a key indicator of harvest efficiency, was significantly improved under the 50% PRI with PBZ compared to traditional irrigation without PBZ application (Fig. 4c). This indicates a beneficial shift in biomass partitioning towards the marketable yield. Similar improvements in crop quality and water use efficiency under 50% PRI have been reported for other species, such as potato^51^. Therefore, when addressing future challenges of water scarcity, establishing a crop-specific long-term water management strategy that leverages synergistic combinations of deficit irrigation techniques and plant growth regulators like paclobutrazol is crucial.

### PBZ and PRI effects on osmotic adjustment and photosynthetic resilience

The interaction between irrigation method and paclobutrazol (PBZ) concentration significantly influenced the physiological and biochemical responses of cauliflower plants, as evidenced by the data in Table 3; Fig. 5. Paclobutrazol demonstrated a pronounced, concentration-dependent effect on increasing chlorophyll content, with the highest values recorded at 75 ppm^49,52,53^. The significant interaction for chlorophyll content, visible in Fig. 5a, indicates that PBZ’s efficacy was dependent on the irrigation regime. While traditional drip irrigation with 75 ppm PBZ yielded the absolute highest values, PBZ application under 50% PRI substantially mitigated the chlorophyll reduction typically caused by water stress^48,52,54^. This enhancement can be attributed to PBZ’s role in protecting chloroplast integrity and promoting chloroplast clustering near cell walls^32,55^. Furthermore, PBZ is known to delay senescence and slow chlorophyll degradation by modulating cytokinin and antioxidant levels, thereby maintaining photosynthetic capacity under stress conditions^52,56^. In contrast to the patterns for chlorophyll, the parameters of leaf cell sap TSS and osmotic pressure revealed a distinct plant response linked to osmotic adjustment. The 50% PRI strategy itself consistently induced higher TSS and osmotic pressure compared to full irrigation (Table 4), representing a fundamental acclimation mechanism where plants accumulate compatible solutes like sugars, proline, and ions to maintain cell turgor and physiological function under drought conditions^57–60^. Paclobutrazol application amplified this response. As shown in Fig. 5b **and c**, the 50 ppm and 75 ppm PBZ treatments under 50% PRI achieved levels of osmotic adjustment that were similar to or greater than those observed under traditional drip irrigation at the same PBZ concentrations, with the 75 ppm concentration under PRI triggering the most intense effect. Concurrently, PBZ enhances resilience by reducing vegetative growth, potentially redirecting carbon towards osmotically active solutes^61^. The combination with PRI optimizes osmotic adjustment. The higher TSS and osmotic pressure under 50% PRI compared to traditional irrigation at similar PBZ levels (Table 3) likely result from active osmoregulation driven by PRI stress signals and PBZ’s metabolic shift rather than passive dehydration^54,62^. This solute accumulation supports metabolic function under water stress.

### Stability of cauliflower yield and water productivity under PBZ and PRI

The yield results revealed the higher performance ofthe traditional drip irrigation method with increasing concentrations of paclobutrazol (Fig. 6a). Moreover, the results revealed an increase in cauliflower production with increasing paclobutrazol concentration. The yield reduction under 50% PRI with 75 ppm paclobutrazol compared to traditional drip irrigation with 0 ppm paclobutrazol was 1.06% and 1.75% for the two respective seasons. This value is very close to the yield under traditional drip irrigation with 0 ppm paclobutrazol. Some studies^63–65^ have shown that plant nutrient uptake is maximized during partial root-zone irrigation (PRI 50%). The reason for this is that plants work to form new roots under partial root-zone irrigation (50% PRI). This leads to smooth absorption of nutrients from the soil and soil water in the root zone. Moreover, paclobutrazol spraying increased the resistance of cauliflower plants to water deficit under 50% PRI, as soil moisture content was maintained within available water limits. Our results demonstrate that paclobutrazol (PBZ) enhanced cauliflower drought resistance under 50% PRI by conserving soil moisture, despite reducing root biomass (Table 1). The conserved moisture, as shown by the higher soil water content in treated plots (Fig. 2), was maintained at levels sufficient to meet plant demand and prevent wilting, thereby mitigating water stress. This adaptation prioritizes water use efficiency and reproductive development over expansive vegetative growth, ultimately supporting yield stability under water deficit. The efficacy of partial root-zone irrigation in maintaining yield quality under water deficit is well established in other crops, such as potato, where it was shown to produce higher-quality tubers compared to other deficit irrigation methods^51^. Furthermore, the general water-saving potential of this approach was recently corroborated by Idris et al.^66^ reporting that the PRD method achieved a 25% reduction in water use without yield penalty. This study significantly advances the evidenceby demonstrating that the combination of 50% PRI and 75 ppm paclobutrazol increasesthe water-saving efficacy of PRI alone, achieving a 50% reduction in irrigation water while maintaining yield equivalent to the fully irrigated control (Fig. 6a). In addition, an increase in irrigation water productivity was achieved by applying 50% PRI with 75 ppm paclobutrazol (49.4 and 49.3%) compared with traditional drip irrigation with 0 ppm paclobutrazol for both seasons (Fig. 6b). Mohamed et al.^67^ reported that the application of paclobutrazol enhances tomato fruit yield and irrigation water productivity. On the other hand, Ahmadi et al.; Rashid et al.; Ahmad et al.^68–70^ reported that irrigation water productivity increased under a 50% PRI for different crops. In a statistical study, Sadras^71^ reported that irrigation water productivity increased by 82% when 50% PRD was applied compared with full irrigation. This supports that the primary benefit of partial root-zone drying deficit irrigation lies in its maximization of irrigation water productivity. As demonstrated in other crops like green beans, applying a 50% water-saving strategy generallyincreases yield and irrigation water productivity. Making it a practice worthy of consideration for regions with limited water availability^72^.

## Conclusions

Water and food security are among of the priorities of countries, especially in light of the increase in population. This study indicates that the combination of partial root-zone irrigation (PRI 50%) and a 75 ppm paclobutrazol (PBZ) application presents a potentially effective water-saving strategy for cauliflower production in sandy loam soils. The key finding is that this approach achieved a statistically equivalent head diameter and total yield to traditional drip irrigation without PBZ while using only half the amount of irrigation water. The success of this strategy is rooted in measurable physiological adaptations. While the treatment reduced vegetative growth, it simultaneously enhanced the plant’s drought resistance. This was supported by a significant increase in leaf osmotic pressure and total soluble solids, which are key indicators of osmotic adjustment that help maintain plant water status and function under stress. The combination of PRI 50% and 75 ppm PBZ improved irrigation water productivity by approximately 49%. This supports the potential of this method for sustainable water use without substantial compromise on marketable yield. Therefore, for agricultural regions facing water scarcity, the adoption of partial root-zone irrigation combined with paclobutrazol application offers a viable and practical strategy to conserve a critical resource. However, since this work was carried out at one field site over two seasons on sandy loam soil using a single cauliflower cultivar and a fixed PRI level (50%), confirming the findings across other soils, climates, cultivars, and PRI regimes would improve their wider applicability.

## Data Availability

All data generated or analyzed during this study are included in this published article.
