# Publisher Correction: Enhancement of cauliflower (Brassica oleracea var. botrytis) water stress resistance using paclobutrazol and partial root-zone irrigation

**DOI:** 10.1038/s41598-026-57600-4

**Published:** 2026-07-01

**Authors:** Ahmed F. El-Shafie, Marwa M. Abdelbaset, Ebtessam A. Youssef, Osama M. Dewedar

**Affiliations:** Water Relations and Field Irrigation Department, National Research Centre, Dokki, Cairo, 12622 Egypt

Correction to: *Scientific Reports* 10.1038/s41598-026-43596-4, published online 05 May 2026

The original PDF version of this Article contained an error Figure 2, where panel b was omitted.

The original Figure 2 and accompanying legend appears below.

**Fig. 2:**
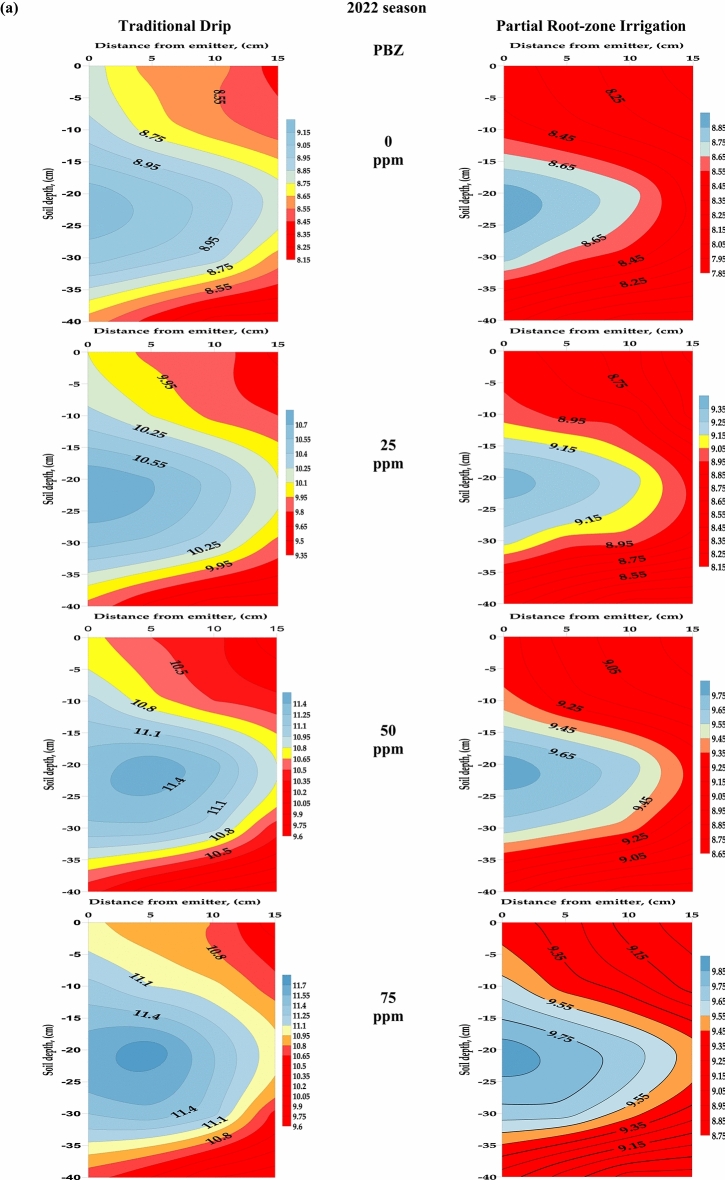
Soil moisture (%) distribution values before irrigation under traditional drip and partial root-zone irrigation (PRI) methods at 55 days after planting after sprayed with different concentrations of paclobutrazol (PBZ) (0, 25, 50, 75 ppm), for **(a)** 2022 and **(b)** 2023 seasons. Measurements were taken at distances from the emitter (0, 5, 10, and 15 cm along the x-axis). Additionally, measurements were recorded at different soil depths (10, 20, 30, and 40 cm along the y-axis). The data were analyzed using SURFER software for contour mapping and spatial modeling. Values are means of 5 replicates ± SE; by Duncan’s new multiple range test at *p* < 0.05.

The original Article has been corrected.

